# Iowa Agricultural College

**Published:** 1893-12

**Authors:** 


IOWA AGRICULTURAL COLLEGE.—VETERINARY DEPARTMENT.

*Degree D. V. M.*

| | |
|---|---|
| A. Bostrom, - - - | Merithan, Iowa. |
| S. H. Guinn, - - - - | Brooklyn, Iowa. |
| Will Pringle, - - - | Maringo, Ill. |
| W. Tanner, - - - - | Butte, Montana. |
| H. L. Stewart, - - - - | Oakley, Iowa. |
| J. L. Williamson, - - - | Jefferson, Iowa. |
| S. T. Miller, - - - | Shelby, Iowa. |

