# Veterinary Colleges; a Correction*Vide, Vol. xiv, No. 6, Fol. 412.

**Published:** 1894-01

**Authors:** James R. Waugh


### VETERINARY COLLEGES.*

IOWA STATE AGRICULTURAL COLLEGE—VETERINATY DEPARTMENT.†

*Degree*—DOCTOR OF VETERINARY MEDICINE, D.V.M.

IOWA VETERINARY COLLEGE.

*Degree*—DOCTOR OF VETERINARY SCIENCE, D.V.S.

### CORRECTION.

BY JAMES R. WAUGH, V.S.

The JOURNAL erred in the December number by crediting the graduates of the Iowa Veterinary College, Des Moines, Iowa, to the Veterinary Department of the Iowa Agricultural College, Ames, Iowa.

The Iowa Veterinary College is a two-year school with a session of six months each year, and grants the degree D.V.S. (Doctor of Veterinary Science); while the Veterinary Department of the Iowa Agricultural College is a three-year school, with a session of nine months each year, and grants the degree D.V.M. (Doctor of Veterinary Medicine). The first named is an autumn and winter school, while the second named is a spring, summer and autumn school. Veterinary students who attend short winter sessions at college would derive great benefit from a course in a summer school.

---

THE KANSAS CITY VETERINARY COLLEGE.

*Degree*—DOCTOR OF VETERINARY SCIENCE, D.V.S.*

Y. H. Wattles, D.V.S., the Secretary and Treasurer of the Kansas City Veterinary College, makes this correction, and adds: "I will also state that, while we see no reason for changing the degree, if the Colleges unite on a uniform degree for all American schools, we will accept the change."

* Vide, Vol. xiv, No. 6, Fol. 412.
† List of graduates for 1893 will appear in the February JOURNAL.

